# Antiviral activity of aspirin against RNA viruses of the respiratory tract—an in vitro study

**DOI:** 10.1111/irv.12421

**Published:** 2016-09-22

**Authors:** Bernadette Glatthaar‐Saalmüller, Kerstin H. Mair, Armin Saalmüller

**Affiliations:** ^1^ Labor Dr. GlatthaarOchsenhausenGermany; ^2^ Department of PathobiologyInstitute of ImmunologyUniversity of Veterinary Medicine ViennaWienAustria

**Keywords:** acetylsalicylic acid, antiviral activity, aspirin, influenza, plaque‐reduction assay, rhinoviruses

## Abstract

**Aim:**

Aspirin (acetylsalicylic acid) has been used for more than 115 years in medicine. Research exists to show that aspirin has antiviral effects in vitro*,* for example, by blocking influenza virus propagation via NF‐κB inhibition when used at high concentrations and short‐term incubation steps. The aim of this study was to confirm the antiviral activity of aspirin against influenza virus and further elucidate the activity of aspirin against other respiratory viruses.

**Methods:**

Tests to detect antiviral activity were performed using plaque‐reduction assays. Aspirin was administered to the virus‐infected cell cultures one hour after infection. Prior to these assays, the non‐cytotoxic concentrations of aspirin on cells used for propagation of the respective viruses were determined.

**Results:**

Aspirin was found to be highly effective against influenza A H1N1 virus. The antiviral activity against further respiratory RNA viruses was less distinct. Respiratory syncytial virus was minimally inhibited. However, the activity of aspirin against rhinoviruses was more pronounced. Aspirin demonstrated antiviral activity against all human rhinoviruses (HRV), but the effect on members of the “major group” viruses, namely HRV14 and HRV39, was greater than on those of the “minor group,” HRV1A and HRV2.

**Conclusions:**

These data demonstrate a specific antiviral activity of aspirin against influenza A virus and HRV. The mode of action against rhinoviruses is still unknown and requires further investigation, as does the possibility of aspirin being effective in vivo to treat the common cold.

## 1 Introduction

Acetylsalicylic acid (ASA), better known for more than 115 years under its trade name of aspirin, was characterised in the past as an active component with a multitude of actions. It is often used to relieve minor aches and pains,^1^ to reduce fever 2, 3 and also as an anti‐inflammatory medication.^4^ Furthermore, aspirin blocks the formation of thromboxane A2 in platelets producing an inhibitory effect on platelet aggregation. Aspirin, therefore, has a positive influence on the prevention of blood clots responsible for heart attacks and strokes.^5^, ^6^, ^7^, ^8^ One basic mechanism in the mode of action of aspirin is the inhibition of prostaglandin synthesis due to the irreversible inactivation of cyclooxygenase 1 and 2 (COX‐1/‐2).^9^, ^10^, ^11^, ^12^ In addition to this primary mechanism, at least three additional modes of action have been described for aspirin: uncoupling of oxidative phosphorylation in hepatic mitochondria,^13^ induction of NO radicals responsible for a decrease in inflammation 14 and some modulation of signalling through transcription factor NF‐κB 15, 16, 17 which plays a central role in many biological processes. Although much research has been carried out on the modes of action of this drug, information regarding activity against infectious agents, for example viral pathogens, is sparse. Reports exist describing antiviral activity against hepatitis C virus RNA and protein expression through COX‐2 signalling pathways.^18^, ^19^ Reactivity against varicella zoster viruses, as well as cytomegalovirus infections, has also been reported.^20^, ^21^, ^22^ Furthermore, antiviral activity against influenza A viruses was postulated to be based on the modulation of the NF‐κB‐pathway.^23^, ^24^ Nevertheless, reports exist which describe an NF‐κB‐independent pathway of inhibition of viral replication with particular respect to flaviviruses 25 and hepatitis C virus.^26^

Here, we present an in vitro study confirming the antiviral effect of aspirin against influenza viruses and investigating the antiviral activity of the drug on a broad panel of human pathogenic viruses, primarily involved in infections or in “superinfections” of the upper respiratory tract. The dose‐dependent antiviral potency of aspirin against a variety of viral candidates was tested in vitro with plaque‐reduction assays using a “therapeutic treatment” method by adding serial dilutions of aspirin to the virus‐infected cell cultures one hour after infection.

The following studies included two human DNA viruses: adenovirus (Adeno 5) and herpes simplex virus (HSV‐1); several RNA viruses: two enveloped RNA viruses, human influenza A (FluA H1N1) and human respiratory syncytial virus (RSV); and two non‐enveloped RNA viruses: Coxsackie virus (CA9) and human rhinoviruses (HRV). Furthermore, we determined the influence of aspirin against two HRV subtypes from both the “minor group” (HRV1A, HRV2) and the “major group” (HRV14, HRV39).

## 2 Material and Methods

### 2.1 Test substance

Test substance was acetylsalicylic acid (ASA, aspirin), molecular mass: 180.16 g/L (1 mol/L) ≥99% crystalline (Sigma‐Aldrich, Munich, Germany (A5376), batch 2014). Aspirin was dissolved in a concentration of 180 mg/mL in 70% ethanol (1M stock solution; Merck, Darmstadt, Germany). This solution was diluted with cell culture medium (Hank's/Earle's minimal essential medium, MEM, PAN‐Biotech GmbH, Aidenbach, Germany) resulting in a 10 mmol/L working solution containing 0.7% ethanol. For the determination of the highest non‐cytotoxic concentrations of aspirin, the working solution was adjusted to pH 7.4 and thereafter titrated in log_2_ dilutions to the respective cell cultures. For the determination of the cytotoxicity of aspirin, the influence of at least 12 serial dilutions was examined.

### 2.2 Reference drugs

To verify the test systems, Ribavirin^®^ (1‐beta‐d‐ribofuranosyl‐1,2,4‐triazole‐carboxamide; Abcam Biochemicals, Cambridge, UK) 27 was included in the assays for the detection of antiviral activity against RNA viruses at concentrations between 5 and 20 μg/mL. Acyclovir^®^ (9‐2‐hydroxyethoxymethylguanine; Sigma‐Aldrich, Wien, Austria)^28^, ^29^ was included in assays with HSV‐1 at a concentration of 2.5 μg/mL. In assays with adenovirus, a composition of plant‐derived substances with the trade name “Sinupret^®^” (Bionorica SE, Neumarkt, Germany) was included as an internal standard.^30^

### 2.3 Cells and viruses

Human influenza A virus strain, influenza A Chile 1/83 H1N1 (FluA H1N1), respiratory syncytial virus, strain Long (RSV), Coxsackie virus subtype A9 (CA9), adenovirus C subtype 5 (Adeno 5) and herpes simplex type 1 (HSV‐1) were obtained from the former Department of Medical Virology and Epidemiology of Viral Diseases of the Hygiene Institute at the University of Tübingen, Germany.

Human rhinovirus B subtype 14 (HRV14) was obtained from the Institute for Virology at the Friedrich‐Schiller‐University, Jena, Germany. Human rhinoviruses HRV‐A1, HRV‐A subtype 2 (HRV2) and HRV‐A subtype 39 (HRV39) were purchased from the American Type Culture Collection (ATCC, HRV‐A1, ATCC‐VR‐1559; HRV2, ATCC‐VR‐482; HRV39, ATCC‐VR‐340). FluA H1N1 was grown on Madin–Darby canine kidney (MDCK) cells with serum‐free MEM (PAN‐Biotech) containing 1 μg/mL of trypsin (Sigma‐Aldrich), 2 mmol/L of l‐glutamine (PAN‐Biotech), 100 U/mL of penicillin (PAN‐Biotech) and 0.1 mg/mL of streptomycin (PAN‐Biotech). RSV, HSV‐1 and Adeno 5 were propagated on human epithelial cells (HEp‐2); HRV1A, HRV2, HRV14 and HRV39 on HeLa cells; and CA9 on buffalo green monkey (BGM) cells, in MEM containing 2% (v/v) foetal calf serum (PAN‐Biotech), 25 mmol/L MgCl_2_ (Sigma‐Aldrich), 2 mmol/L of l‐glutamine, 100 U/mL of penicillin and 0.1 mg/mL of streptomycin.

To determine virus titres, the respective cells were incubated with serially diluted virus stock solutions for 1 hour at 34°C. After removal of the virus inoculum, cell cultures were overlaid with the respective virus‐specific medium containing agarose (0.1%–0.4%, depending on the virus strain). Analyses of plaques (plaque‐forming unit, PFU) and any cytopathogenic effects (CPE) were performed 3–6 days later. The respective virus titres were calculated as PFU per mL according to the method detailed by Cavalli‐Sforza or with the Spearman–Karber method by the mean infectious dose (log_10_ tissue cell infective dose (TCID)_50_) per mL (Adeno 5).

### 2.4 Cytotoxicity tests

Analyses of the in vitro cytotoxicity of aspirin were performed on physiologically active cells using 3‐(4,5‐Dimethylthiazol‐2‐yl)‐2,5‐diphenyltetrazoliumbromid (MTT) assays,^31^ which are capable of quantifying the activity of mitochondrial enzymes demonstrating a direct correlation between viability and enzyme activity. For the determination of the limits of toxic concentrations, cells were cultivated together with varying dilutions (log_2_ dilutions ranging from 10 to 0.01 mmol/L and a final log_10_ dilution to 0.001 mmol/L) of aspirin and the ethanol control at 37°C and 5% CO_2_ for at least 6 days. Cells cultivated in cell culture media alone were used as an additional control (medium control). Analyses with the MTT assay were performed on days 1, 3 and 6 after the addition of aspirin to the cell cultures. For this purpose, cells were incubated for 1 hour with an MTT solution and after dissolution of the formazan crystals in DMSO (Sigma‐Aldrich). The optical density (OD) of the cell culture supernatants was analysed in a photometer at 570 nm (Spectrafluor Tecan, Crailsheim, Germany). In addition, microscopic analyses of the cell monolayers were performed 6 days after the introduction of aspirin. To be classed as a positive result, the following criteria were applied to these assays: altered cell morphology, generation of intracellular vacuoles and the destruction of cell monolayers.

### 2.5 Determination of the cytotoxicity

**Figure 1:**
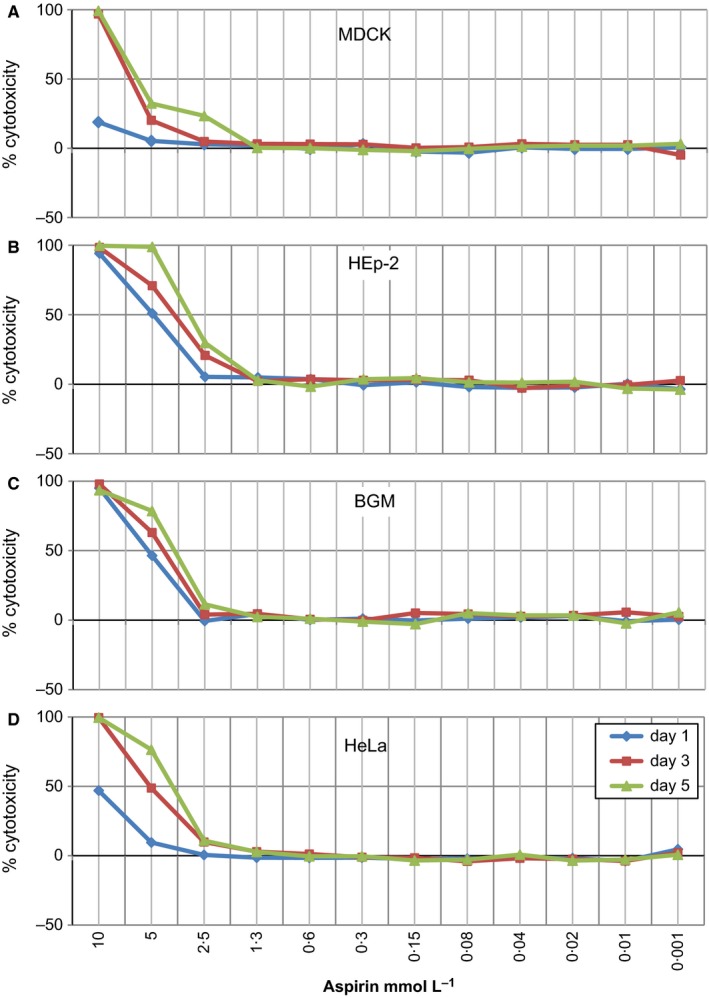
In vitro cytotoxicity of aspirin. In vitro cytotoxicity of aspirin was tested on physiologically active cells used for the propagation of viruses: MDCK (A), HEp‐2 (B), BGM (C) and HeLa (D) over a period of several days. The titration curves show the dose‐dependent cytotoxicity determined using six replicates for each serial dilution for day 1 (blue), day 3 (red) and day 5 (green) for each of the cell lines. The standard deviations in all assays ranged between approximately 2% and 10%. IC50 values were determined graphically and are presented in Table 1

Optical density (OD) values of the non‐treated medium controls were defined as 100% viability. The OD values of the supernatants derived from aspirin‐treated cell cultures were calculated accordingly. A relative toxicity (% cytotoxicity) was determined using six replicates for each serial dilution. Within the respective dose–response curves, the 50% toxicity concentration (IC_50_) was defined graphically (Fig. 1A–D).

### 2.6 Assays for antiviral activity

The antiviral activity of aspirin was measured using plaque‐reduction assays in plaque‐forming units (PFU) for FluA H1N1, RSV, CA9, HSV‐1, HRV1A, HRV2, HRV14 and HRV39 or by analysing CPE for Adeno 5. Cell monolayers were infected with a multiplicity of infection (M.O.I.) between 0.0004 TCID_50_/mL for FluA H1N1, RSV, CA9, HSV‐1, HRV1A, HRV2, HRV14, HRV39 and 10^3^ TCID_50_/mL for Adeno 5 for 1 hour at 34°C. Cell monolayers were then washed and overlaid with agarose (0.1%–0.4% final concentration) containing non‐toxic concentrations of aspirin (ranging from 2 to 0.005 mmol/L). Subsequently, the infected cell cultures were cultivated for 3–6 days until lesions were visible in the cell monolayer (plaques or CPE) of the virus‐infected control group cultivated in medium alone. The cells were then fixed with paraformaldehyde and stained with a crystal violet solution. Non‐stained lesions in the cell monolayer (plaques, CPE) were quantified by an optical evaluation system (ELISpot reader, AID Diagnostika GmbH, Straßberg, Germany). A detailed summary of M.O.I.s, analyses, standards and cultivation times is presented in Table S1.

### 2.7 Calculation of antiviral activity

The quantification of antiviral activity was either carried out by analysing the number of plaques (PFU: FluA H1N1, RSV, CA9, HSV‐1, HRV1A, HRV2, HRV14, HRV39), or the lesions of viral CPE (Adeno 5), with an ELISpot reader. The calculation of the antiviral effect was based on mean values of four replicates (CPE or PFU) derived from at least two or three independent experiments, respectively (for details, see Table S2). A solvent (ethanol) control, which did not differ in its cytotoxicity from the medium control, was included in all assays. The results of the non‐treated virus control groups were defined as 100% infection (0% inhibition), and in vitro effects of the test substance were standardised as relative inhibitory effects. Original data from 6‐well plates for studies with FluA H1N1 and HRV14 are presented in Figs S1 and S2, respectively.

## 3 Results

### 3.1 Determination of in vitro cytotoxicity of aspirin

Prior to the assays determining the antiviral activity of aspirin, its cytotoxic effect on cells used for the antiviral assays had to be determined. The standard solution of aspirin (1 mol/L in 70% ethanol) was, therefore, diluted to a working solution of 10 mmol/L with cell culture medium (MEM). Assays for the detection of non‐cytotoxic concentrations of aspirin began at a concentration of 10 mmol/L aspirin followed by at least 12 serial log_2_ and a final log_10_ dilution step. These dilutions were added to cells which were then cultivated for 6 days. As controls, cells incubated with medium alone (non‐treated controls) or incubated with medium containing 0.7% and 0.35% ethanol were used. MTT assays 31 were performed on days 1, 3 and 5. From these tests, clear cytotoxicity thresholds could be determined. Concentrations of 10 mmol/L aspirin resulted in complete lysis of the cell monolayers and 95% cytotoxicity for all four cell lines in MTT assay (Fig. 1A–D). No toxic effects of aspirin were detectable at concentrations of less than 2.5 mmol/L. Integrating the cytotoxicity ranges analysed over several days (day 1, day 3 and day 5), the IC50 values – indicating 50% toxicity – ranged between 6.25 mmol/L and over 10 mmol/L for MDCK cells, between 3.51 and 5.04 mmol/L for HEp‐2 cells, between 3.96 and 5.41 mmol/L for BGM cells and between 4.06 mmol/L and over 10 mmol/L for HeLa cells. As expected, the ethanol solvent controls at starting concentrations of 0.7% and 0.35% ethanol did not cause any cytotoxic effects. Based on the results of the cytotoxicity tests, it was decided to begin the assays for the detection of antiviral activity at a concentration of 2 mmol/L aspirin for FluA H1N1 and 1 mmol/L aspirin for RSV, CA9, HRV1A, HRV2, HRV14, HRV39, HSV‐1 and Adeno 5. This concentration was then followed by five log_2_ and a final log_10_ dilution of aspirin.

### 3.2 Determination of the antiviral activity of aspirin

**Figure 2:**
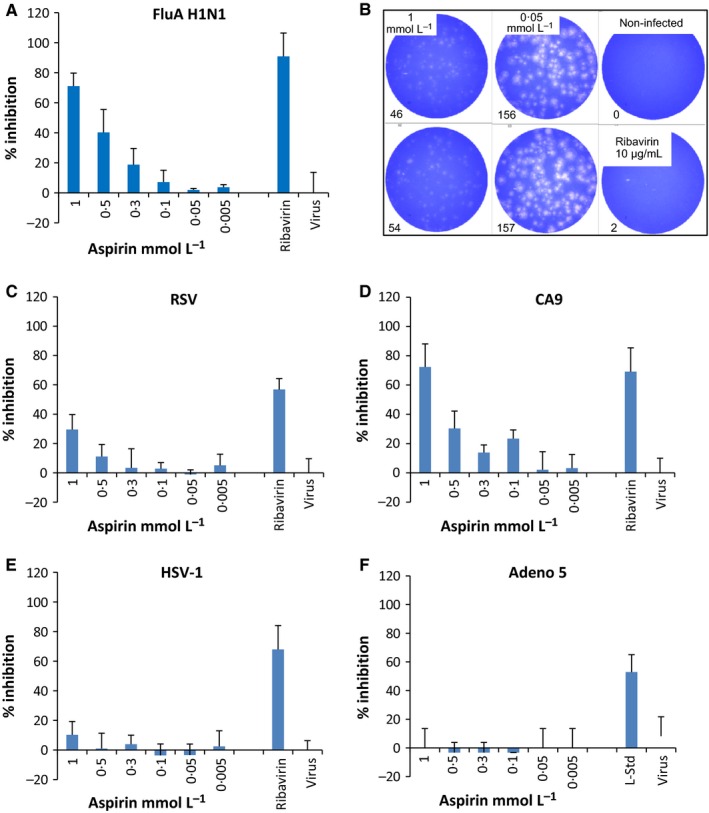
Determination of a dose‐dependent antiviral activity of aspirin against RNA viruses (FluA A; RSV, C; CA9, D) and DNA viruses (HSV‐1, E; Adeno 5, F). A semi‐quantification step was performed using plaque‐reduction assays (for FluA, RSV, CA9, HSV‐1) or with analyses of cytopathogenic effect and the quantification of viral proteins (Adeno 5). X‐axes show the titration of aspirin. Virus controls (without test substance) as well as positive controls are included in the figure: Ribavirin^®^ (5–20 μg/mL) against FluA (A, B), RSV (C), CA9 (D) and Acyclovir^®^ for HSV‐1 (E) and Sinupret^®^ 30 (7.5 μg/mL, laboratory standard, l‐Std) against Adeno 5 (F). The figures present the percentage inhibition of the infectivity of the aspirin‐treated cell cultures (MDCK/FluA; HEp‐2/RSV; HEp‐2/HSV‐1; HEp‐2/Adeno 5; BGM/CA9) in comparison with the non‐treated virus control (100% infection). Data are derived from four replicates of one representative study of two or three different experiments. The standard deviations in all assays ranged between 5% and 15%. The virus doses used for the respective infection experiments (multiplicities of infection, M.O.I.) differed finally between an M.O.I. of 0.0004 (RSV, CA9, HSV‐1) and 10^3^ TCID_50_/mL (Adeno 5)

To test whether aspirin has the ability to inhibit virus replication, cells were infected with FluA H1N1, RSV, HSV‐1, Adeno 5, CA9 and a variety of rhinovirus strains. A subset of the results of the respective analyses are presented in Fig. 2, showing the dose‐dependent antiviral activity against FluA H1N1 (Fig. 2A). Figure 2B illustrates some examples of original study results, presented graphically in Fig. 2A. With respect to antiviral activity against FluA H1N1, aspirin induced a considerable dose‐dependent reduction in virus infection, with a 71.1% reduction being attained at a concentration of 1 mmol/L (Fig. 2A). This antiviral effect decreased at lower aspirin concentrations. For a more detailed illustration of the dose‐dependent antiviral activity of aspirin against FluA H1N1, photographs of original 6‐well plates are provided in Fig. S1.

In summary, a dose‐dependent effect of aspirin against FluA H1N1 could be demonstrated in all experiments, with EC50 values between 0.66 and 0.87 mmol/L (Table 1 and Table S2). In Fig. 2C–F, the dose‐dependent antiviral activity against RSV (C), CA9 (D), HSV‐1 (E) and Adeno 5 (F) for one (of 2 or 3) representative experiments is illustrated. The antiviral activity of aspirin was determined using plaque‐reduction assays for RSV, CA9 and HSV‐1 32 or in analyses of a cytopathogenic effect (CPE) for Adeno 5. The addition of aspirin to the cell cultures infected with the DNA viruses HSV‐1 and Adeno 5 did not lead to any antiviral activity (Fig. 2E,F, respectively). However, a low level of antiviral activity could be demonstrated against RSV, an enveloped RNA virus, at a concentration of 1 mmol/L aspirin (Fig. 2C). A pronounced dose‐dependent effect of aspirin was visible for infections with CA9 (Fig. 2D). A concentration of 1 mmol/L of aspirin induced a 72.3% inhibition of viral plaques, and further dilutions of 0.5 and 0.3 mmol/L continued to lead to a visible negative effect on CA9 virus replication in vitro. Data regarding the antiviral activity of aspirin are summarised in Table 1, presenting the effective concentrations (EC50, mmol/L) of aspirin that led to a 50% reduction in virus replication. Data derived from additional experiments are presented in Table S2.

**Table 1:** EC50/IC50

Virus strain	DNA/RNA viruses enveloped/non‐enveloped	Cells for propagation	aEC50 values mmol/L	bIC50 values mmol/L
Influenza A H1N1 (FluA H1N1)	RNA enveloped	MDCK	0.66	6.25 to >10
Respiratory syncytial virus (RSV)	RNA enveloped	HEp‐2	≥1	3.51–5.04
Herpes Simplex Virus (HSV‐1)	DNA enveloped	HEp‐2	>1	3.51–5.04
Adenovirus (Adeno 5)	DNA non‐enveloped	HEp‐2	>1	3.51–5.04
Coxsackie virus (CA9)	RNA non‐enveloped	BGM	0.98	3.96–5.41
Rhinovirus (HRV)	RNA non‐enveloped	–	–	–
HRV1A	Minor group	HeLa	≥1	4.06 to ≥10
HRV2		HeLa	0.69	4.06 to ≥10
HRV14	Major group	HeLa	0.21	4.06 to ≥10
HRV39		HeLa	0.1	4.06 to ≥10

a EC50, effective concentration (antiviral activity).

b IC50, inhibitory concentration (cytotoxicity, range between day 1, day 3 and day 5).

**Figure 3:**
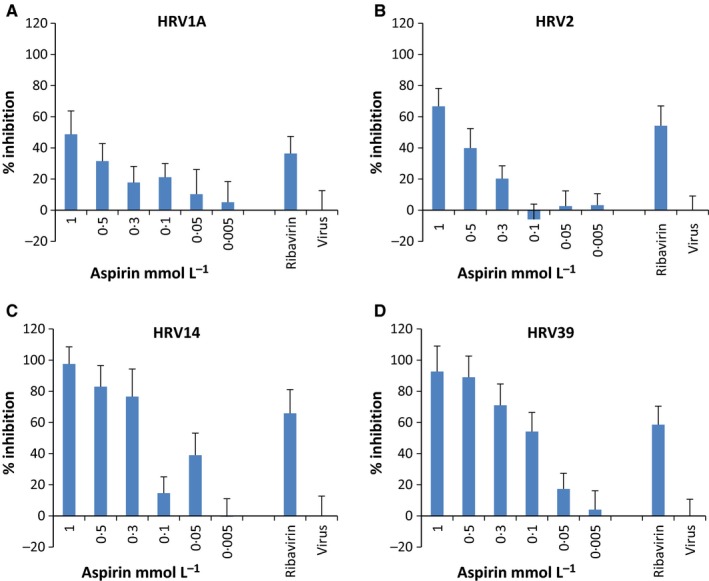
Determination of the antiviral activity of aspirin against human rhinoviruses: Plaque‐forming units per mL (PFU/mL) were determined for quantification of the antiviral activity against HRV1A (A), HRV2 (B), HRV14 (C) and HRV39 (D). The figures present the percentage inhibition of the infectivity of the aspirin‐treated cell cultures (HeLa cells) in comparison with the non‐treated virus control (100% infection). Ribavirin^®^ (10–20 μg/mL) served as internal positive control. Data derived from four replicates of one representative study of three different experiments. The standard deviations in all assays ranged between 7% and 18%. All viruses were used with an M.O.I. of 0.0004

The positive results from the analyses of the antiviral activity of aspirin against the RNA viruses, FluA H1N1 and CA9, led to the hypothesis that aspirin might have a specific antiviral activity against such viruses. Therefore, this putative antiviral effect was tested on additional non‐enveloped RNA viruses: rhinoviruses. The results of these analyses are presented in Fig. 3, showing the dose‐dependent antiviral activity against HRV1A (Fig. 3A), HRV2 (Fig. 3B), HRV14 (Fig. 3C) and HRV39 (Fig. 3D) for one (of 3) representative experiment. Data derived from the remaining experiments are presented in Table S2. The antiviral activity of aspirin against HRV1A and HRV2 resulted in reductions of 48.7% for HRV1A (Fig. 3A) and 66.7% for HRV2 (Fig. 3B) at the highest concentration comparable to reduction levels achieved against CA9 (Fig. 2D). This antiviral activity was dose‐dependent, with EC50 values determined for HRV1A and HRV2 at concentrations of ≥1 and 0.69 mmol/L, respectively (Table 1). The efficacy of aspirin against HRV14 and HRV39 was particularly noteworthy (Fig. 3C,D, respectively). Against both virus strains, the addition of aspirin, at its highest concentration of 1 mmol/L, was shown to lead to a >90% reduction in plaques. This dose‐dependent activity ceased at the lowest concentration of 0.01 mmol/L aspirin for HRV14 (Fig. 3C) and at 0.05 mmol/L for HRV39 (Fig. 3D), with EC50 values of 0.21 mmol/L for HRV14 and 0.1 mmol/L for HRV39 (Table 1). Overall, these results demonstrated that aspirin exhibited a highly specific dose‐dependent antiviral activity against the enterovirus CA9 and against all investigated rhinoviruses. The highest efficacy could be detected against rhinoviruses belonging to the “major group” of the rhinovirus family, namely HRV14 and HRV39.

For a more detailed illustration of the dose‐dependent antiviral activity of aspirin against HRV14, photographs of original 6‐well plates are displayed in Fig. S2.

The dose‐dependent antiviral activity of aspirin against all viruses studied is summarised with EC50 values in Table 1. To enable a better understanding of the antiviral potential of aspirin, this table also contains the IC50 values determining the 50% cytotoxicity value of aspirin with respect to the cell lines used for virus propagation and the antiviral assays.

Further details of the dose‐dependent antiviral activity of aspirin, including controls and additional experiments (study 1–3), are summarised in Table S2.

## 4 Discussion

In all experiments, aspirin showed a considerable dose‐dependent antiviral activity against CA9, HRV1A, HRV2 and substantial activity against FluA H1N1, HRV14 and HRV39. With respect to rhinoviruses in particular, further research is required into the variation in responses observed between “major” and “minor group” strains.

The basic mechanism for this potent antiviral activity against members of the Picornaviridae and Orthomyxoviridae families is still not clear. One might argue that rhinoviruses are known to be extremely pH‐sensitive and that the apparent antiviral effect of aspirin may be simply based on a reduction in pH in cell cultures. To counteract this claim, we carefully monitored pH throughout our experiments and used a very stable buffer system with phenol red as a highly sensitive indicator. In addition, this possible acid effect has also been excluded by others studying the antiviral activity of aspirin against, for example varicella zoster virus and human cytomegalovirus.^20^, ^21^, ^22^ Another argument against a technical artefact is the fact that, in addition to the pH‐sensitive rhinoviruses, the pH‐insensitive enterovirus Coxsackie A9 and influenza H1N1 were also affected by aspirin treatment. In vivo experiments with influenza A viruses have suggested the involvement of the NF‐κB‐pathway 24 and differential regulation of influenza virus RNA synthesis by NF‐κB.^33^ Whether the NF‐κB‐pathway is involved in the reactivity against Picornaviridae or whether other cellular mechanisms play a role has yet to be verified. This remains an important question because Liao et al. 25 were able to demonstrate that blocking of the replication of flaviviruses was independent of blocking NF‐κB activation. These authors discussed the involvement of p38 MAP‐kinase activation and correlated this in vitro activation with a suppression of flavivirus‐induced apoptosis.^25^ In the case of hepatitis C virus (HCV), an effect on the cellular receptor claudin‐1 is postulated 26 leading to an effect on virus entry. However, this does not appear to be the sole effect of aspirin against HCV as others have described an additional effect on the inducible nitric oxide synthase (iNOS), which seems to be implicated in the antiviral activity of aspirin in HCV‐expressing cells.^34^ The effect of aspirin on the iNOS expression by downregulating the promoter activity, mRNA and protein expression levels also appears to be only part of the story, but one which correlates with the known COX‐2 inhibiting activity of aspirin.^34^ Possible speculation about the mode of action can be made about the fact that, in our experiment, only RNA viruses were the target of the antiviral activity of aspirin. This suggests at least some level of specificity. Further research will be required to confirm this apparent effect, and it should be noted that the results obtained here appear to contrast with the data derived from the antiviral activity studies carried out against DNA viruses: varicella zoster virus and HCMV.^20^, ^21^, ^22^

Another important point is the in vivo efficacy of aspirin for the treatment of influenza and other respiratory viruses. One might argue that the effective concentrations (EC50) leading to antiviral activity presented here in in vitro experiments are too high to expect a potential effect in vivo. However, the EC50 values in all cell cultures ranged from 0.69 mmol/L against HRV2 to 0.66 mmol/L for influenza A and to 0.21 and 0.1 for HRV14 and HRV39 infections, respectively. These levels are within the range of plasma levels described for aspirin; anti‐inflammatory plasma levels have been described between 1.5 and 2.5 mmol/L for the total and 0.15–0.60 mmol/L for free salicylate.^35^ Furthermore, it is interesting to note that toxic concentrations in vivo have been described starting at a 2 mmol/L plasma salicylate level. When we compare these toxicity data with our IC50 data, similarities are obvious, with IC50 data of 3.51 mmol/L and 4.06 mmol/L for Hep‐2 and HeLa cells, respectively.

At present, we can only speculate on clinical results, but a comparison of our data with the literature appears to support the possibility of potential antiviral activity against rhinoviruses in vivo. As this question was not the focus of our in vitro studies, additional research is required in this area.

## Conflict of Interests

This study was supported by Bayer Vital GmbH, Leverkusen, Germany.
