# Supplementary material for: Antiviral activity of aspirin against RNA viruses of the respiratory tract—an in vitro study
Source: Influenza Other Respir Viruses. 2016 Sep 22;11(1):85–92. doi: 10.1111/irv.12421 (PMC5155651; doi:10.1111/irv.12421)
Supplement: Supplementary file 1 [file IRV-11-85-s001.pptx]

## Slide 1
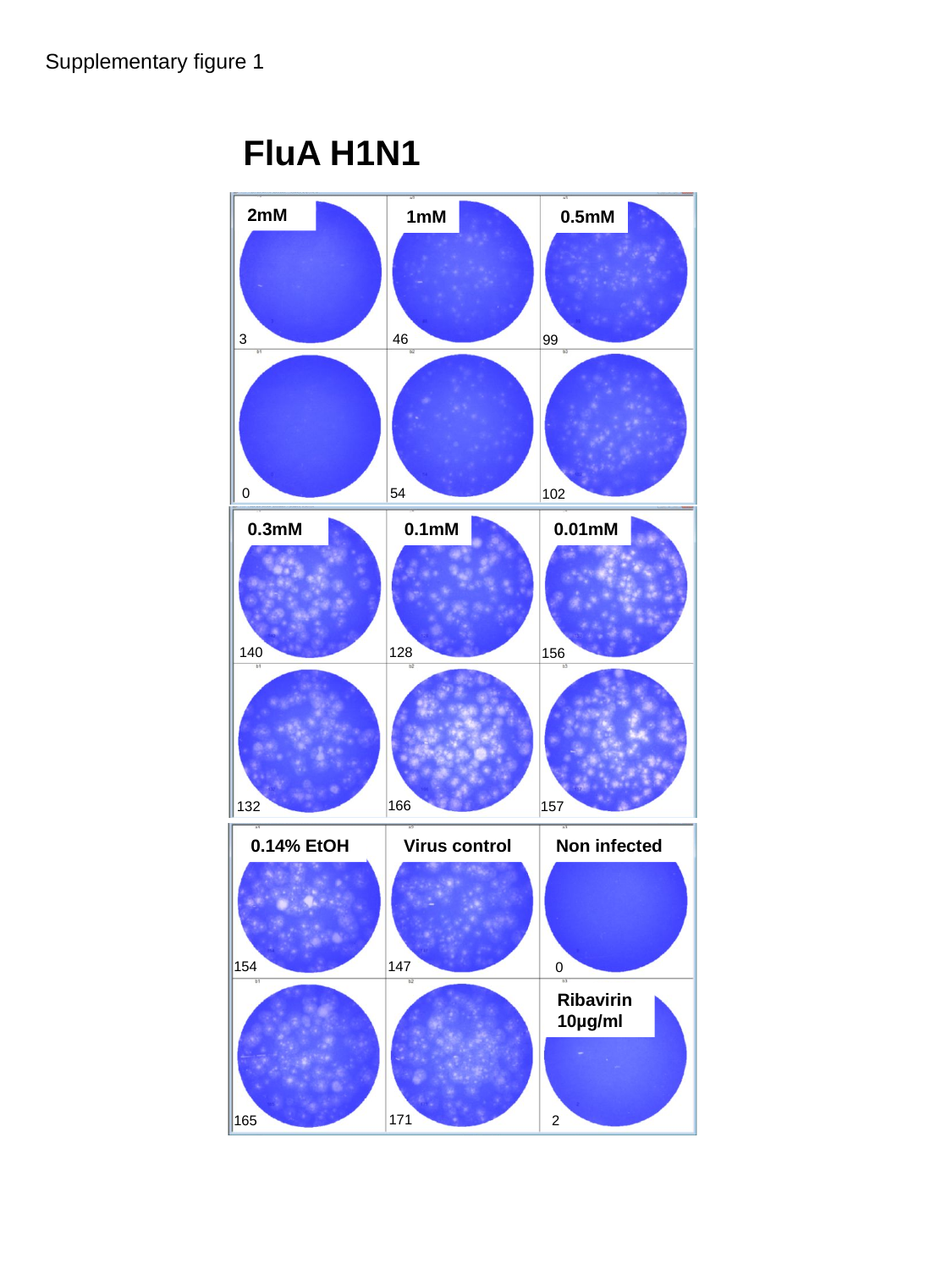

Supplementary figure 1
FluA H1N1
2mM
1mM
0.5mM
46
3
99
54
0
102
0.3mM
0.1mM
0.01mM
128
140
156
166
132
157
0.14% EtOH
Virus control
Non infected
147
154
0
Ribavirin
10µg/ml
171
165
2
