# Supplementary material for: Antiviral activity of aspirin against RNA viruses of the respiratory tract—an in vitro study
Source: Influenza Other Respir Viruses. 2016 Sep 22;11(1):85–92. doi: 10.1111/irv.12421 (PMC5155651; doi:10.1111/irv.12421)
Supplement: Supplementary file 2 [file IRV-11-85-s002.pptx]

## Slide 1
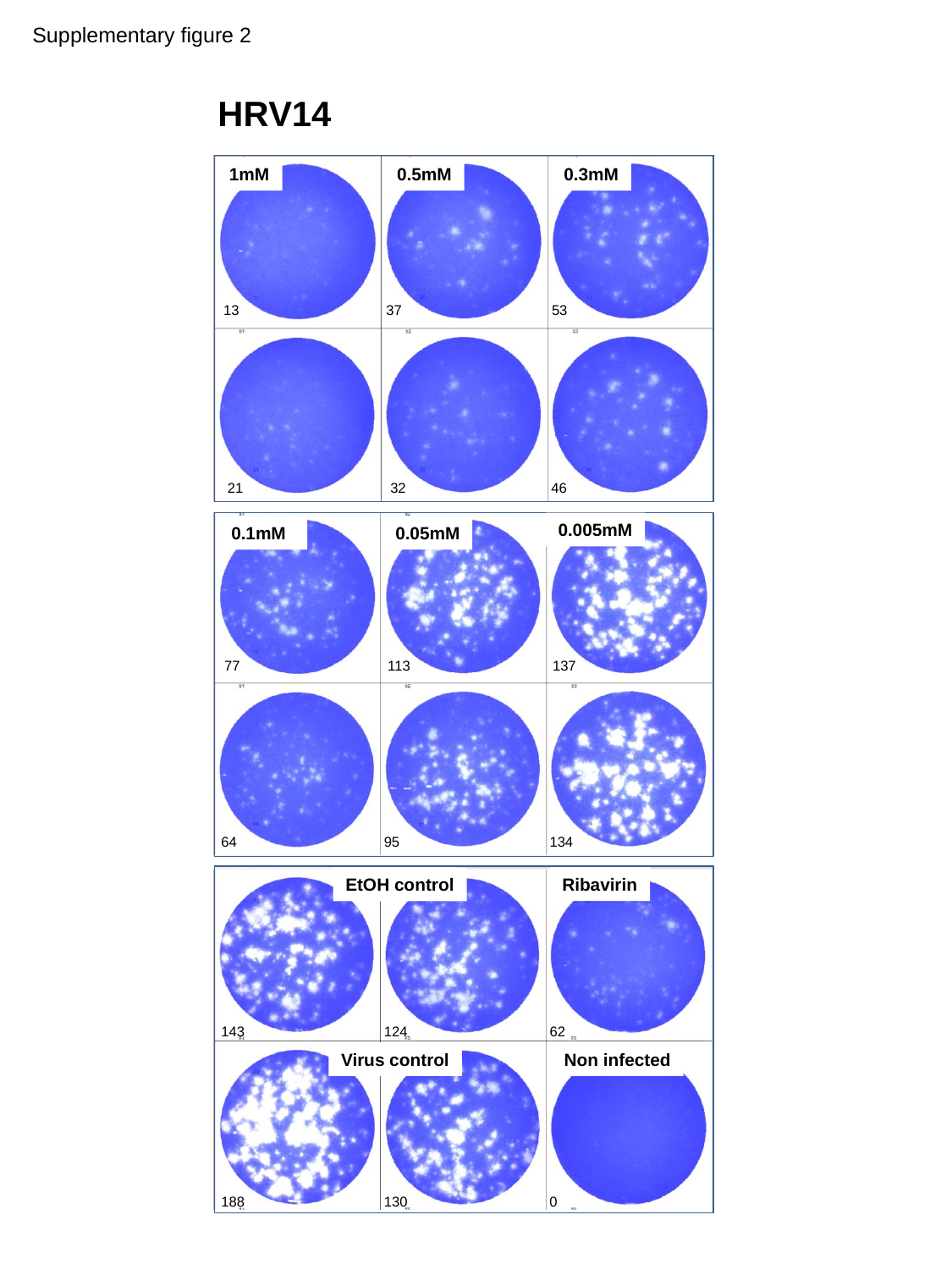

Supplementary figure 2
HRV14
1mM
0.5mM
0.3mM
13
53
37
21
46
32
0.005mM
0.1mM
0.05mM
77
137
113
64
134
95
Ribavirin
EtOH control
143
62
124
Virus control
Non infected
188
0
130
