# Supplementary material for: Antiviral activity of aspirin against RNA viruses of the respiratory tract—an in vitro study
Source: Influenza Other Respir Viruses. 2016 Sep 22;11(1):85–92. doi: 10.1111/irv.12421 (PMC5155651; doi:10.1111/irv.12421)
Supplement: Supplementary file 3 [file IRV-11-85-s003.docx]

**Supplementary table 1**

| Virus strain | cell-  culture | Anti-viral study/ M.O.I.#  PFU/ml / TCID50/ml | | Analyses /  day p.i. | *Positive controls  **internal standard |
| --- | --- | --- | --- | --- | --- |
| FluA H1N1 | MDCK | Study 1-3 | 0.0004 | Plaque reduction  day 3 p.i. | Ribavirin (5-10µg/ml) |
| RSV | HEp-2 | Study 1-2 | 0.0004 | Plaque reduction  day 6 p.i. | *Ribavirin  (5µg/ml) |
| CA9 | BGM | Study 1-2 | 0.0004 | Plaque reduction  day 3 p.i. | *Ribavirin  (20µg/ml) |
| HSV-1 | HEp-2 | Study 1-2 | 0.0004 | Plaque reduction  day 4 p.i. | *Acyclovir  (25µg/ml) |
| Adeno 5 | HEp-2 | Study 1-2 | 0.004 | CPE + ELISA  day 5 p.i. | **plant-derived control substance (7,5µg/ml) |
| HRV1A | HeLa | Study 1-3 | 0.0004 | Plaque reduction day 4 p.i. | *Ribavirin  (15-20µg/ml) |
| HRV2 | HeLa | Study 1-3 | 0.0004 | Plaque reduction day 4 p.i. | *Ribavirin  (15-20µg/ml) |
| HRV14 | HeLa | Study 1-3 | 0.0004 | Plaque reduction day 3 p.i. | *Ribavirin  (10-15µg/ml) |
| HRV39 | HeLa | Study 1-3 | 0.0004 | Plaque reduction day 5 p.i. | *Ribavirin  (10-15µg/ml) |

^#^M.O.I. (multiplicity of infection) of 0.0004 (equivalent 1-1.7 x 10^2^ PFU/ml) and 0.004 (equivalent 10^2^-10^3^ TCID50/ml) depending on the cell count.
