# Supplementary material for: Antiviral activity of aspirin against RNA viruses of the respiratory tract—an in vitro study
Source: Influenza Other Respir Viruses. 2016 Sep 22;11(1):85–92. doi: 10.1111/irv.12421 (PMC5155651; doi:10.1111/irv.12421)
Supplement: Supplementary file 4 [file IRV-11-85-s004.docx]

Supplementary table 2

| Virus | study | % inhibition | | | | | | | | | | |
| --- | --- | --- | --- | --- | --- | --- | --- | --- | --- | --- | --- | --- |
|  |  | aspirin mM | | | | | | | virus controls | | EC50 mM | IC50 mM |
|  |  | 2 | 1 | 0.5 | 0.3 | 0.1 | 0.05 | 0.005 | reference | solvent |  |  |
| FluA H1N1 | **1** | **99.2** | **69.4** | **33.5** | **25.4** | **4.2** | **n.d** | **0.2** | **97.4** | **0.3** | **0.66** | 6.87 |
|  | #2 | 92.3 | 55.8 | 28.1 | 5.4 | 3.4 | n.d | -4.9 | 58.5 | 4.8 | 0.72 |  |
|  | 3 | n.d | 71.1 | 48.2 | 18.8 | 7.2 | 2.0 | 3.8 | 90.9 | 5.4 | 0.87 |  |
| RSV | **1** | **n.d** | **29.5** | **11.1** | **3.4** | **2.8** | **-1.1** | **5.1** | **56.8** | **2.5** | **≥1** | 3.51 |
|  | 2 | n.d | 25.4 | 3.7 | -5.9 | 3.2 | -0.5 | 3.2 | 67.9 | -4.4 | ≥1 |  |
| CA9 | **1** | **n.d** | **72.3** | **30.3** | **13.8** | **23.4** | **2.1** | **3.2** | **69.1** | **2.3** | **0.98** | 4.38 |
|  | 2 | n.d | 67.1 | 28.0 | 26.4 | 2.8 | -4.9 | 3.3 | 82.5 | -3.7 | 0.78 |  |
|  | 3 | n.d | 62.9 | 27.9 | 1.4 | 6.7 | 0.9 | -2.3 | 70.0 | 0.9 | 0.81 |  |
| HSV-1 | **1** | n.d. | **10.2** | **1.0** | **3.9** | **-3.7** | **-3.4** | **2.4** | **67.9** | **2.33** | **>1** | 3.95 |
|  | 2 | n.d | 9.0 | -0.8 | 0.1 | -0.9 | 0.2 | 0.8 | 61.1 | -1.33 | >1 |  |
| Adeno 5 | **1** | n.d | **0.0** | **-3.2** | **-3.2** | **-3.2** | **0.0** | **0.0** | **52.9** | **4.75** | **>1** | 3.51 |
|  | 2 | n.d | 3.2 | -6.5 | 6.5 | 0.0 | 3.2 | 0.0 | 64.5 | -6.19 | >1 |  |
| HRV1A | 1 | n.d | 39.5 | 23.7 | 19.1 | 0.0 | -4.7 | -6.5 | 45.1 | 2.3 | ≥1 | 5.25 |
|  | 2 | n.d | 40.1 | 18.3 | 15.9 | 15.6 | 6.1 | 0.6 | 41.9 | -2.4 | ≥1 |  |
|  | **3** | **n.d** | **48.7** | **31.5** | **17.8** | **21.2** | **10.3** | **5.2** | **36.4** | **4.0** | **≥1** |  |
| HRV2 | **1** | **n.d** | **66.7** | **39.9** | **20.3** | **-5.9** | **2.6** | **3.3** | **54.2** | **-7.2** | **0.69** | 5.25 |
|  | 2 | n.d | 53.9 | 33.9 | 13.9 | 11.7 | 11.1 | -2.8 | 72.2 | -0.6 | 0.88 |  |
|  | 3 | n.d | 57.3 | 45.6 | 18.8 | 13.6 | 6.9 | 0.0 | 35.6 | 7.4 | 0.66 |  |
| HRV14 | **1** | **n.d** | **97.6** | **82.9** | **76.6** | **33.6** | **39.0** | **-0.5** | **65.9** | **2.4** | **0.21** | 4.06 |
|  | 2 | n.d | 95.0 | 83.6 | 51.7 | 48.6 | 24.1 | -2.5 | 52.3 | -3.1 | 0.2 |  |
|  | 3 | n.d | 89.3 | 78.3 | 68.9 | 55.7 | 34.6 | 14.8 | 61.0 | -5.2 | 0.09 |  |
| HRV39 | 1 | n.d | 85.8 | 78.7 | 53.2 | 35.5 | -0.7 | 3.5 | 72.6 | -2.9 | 0.26 | 5.25 |
|  | **2** | **n.d** | **92.7** | **89.0** | **71.0** | **54.2** | **17.3** | **4.0** | **58.6** | **-4.2** | **0.1** |  |
|  | 3 | n.d | 80.8 | 68.2 | 60.3 | 21.0 | 5.2 | 0.6 | 66.8 | 3.3 | 0.25 |  |

EC50 effective concentration (anti-viral activity)

IC50 inhibitory concentration (cytotoxicity)

Studies in **bold** demonstrate data presented graphically in figures 2, 3 and 4

#2 for this FluA H1N1 study aspirin was used in concentrations of 1.5, 0.8, 0.4, 0.2, 0.1, and 0.01mM.

Reference controls: Ribavirin (5-20µg/ml, RNA viruses); Aciclovir (2.5µg/ml, Herpes viruses), plant-derived substance (7.5µg/ml, Adeno 5).

Solvent controls: EtOH (0.07 - 0.14%) in cell culture medium corresponding to the highest concentrations of aspirin (1mM - 2mM) used in the tests respectively did not show any antiviral effects.
